# Development and Recent Advances in Lysine and N-Terminal Bioconjugation for Peptides and Proteins

**DOI:** 10.3390/molecules28031083

**Published:** 2023-01-21

**Authors:** Ajcharapan Tantipanjaporn, Man-Kin Wong

**Affiliations:** State Key Laboratory of Chemical Biology and Drug Discovery, Department of Applied Biology and Chemical Technology, The Hong Kong Polytechnic University, Hung Hum, Hong Kong, China

**Keywords:** bioconjugation, lysine, N-terminal modification, peptide, protein, site-selectivity

## Abstract

The demand for creation of protein diversity and regulation of protein function through native protein modification and post-translational modification has ignited the development of selective chemical modification methods for peptides and proteins. Chemical bioconjugation offers selective functionalization providing bioconjugates with desired properties and functions for diverse applications in chemical biology, medicine, and biomaterials. The amino group existing at the lysine residue and N-terminus of peptides and proteins has been extensively studied in bioconjugation because of its good nucleophilicity and high surface exposure. Herein, we review the development of chemical methods for modification of the amino groups on lysine residue and N-terminus featuring excellent selectivity, mild reaction conditions, short reaction time, high conversion, biocompatibility, and preservation of protein integrity. This review is organized based on the chemoselectivity and site-selectivity of the chemical bioconjugation reagents to the amino acid residues aiming to provide guidance for the selection of appropriate bioconjugation methods.

## 1. Introduction

Bioconjugation has emerged as a powerful synthetic tool to provide multifunctional bioconjugates with diverse applications in chemical biology research. The covalent bond formed by bioconjugation serves as a linkage between biomolecules (e.g., carbohydrates, nucleic acids, viral particles, peptides, and proteins) and other functional molecules (e.g., drugs, polymers, nanoparticles, patterned surfaces, and particulates) to give the desired bioconjugates [1,2,3]. The desirable properties inherited from the bioconjugates allow researchers to exploit them in different research fields, including chemical biology, medicine, materials science, biotechnology, and biophysics [4]. Click Chemistry, the well-established and widely used synthetic method for bioconjugation, has been awarded The Nobel Prize in Chemistry 2022 “for the development of click chemistry and bioorthogonal chemistry”, highlighting the significant contribution of bioconjugation on diverse research areas [5,6,7].

Proteins are an important building block in organisms and are involved in various biological activities. During or after the biosynthesis of proteins, post-translational protein modifications (PTMs) are the key processes to incorporate functional groups to the proteins through covalent bond formation, leading to the change of protein properties [8]. Thus, PTMs, the natural protein modifications, greatly expand the biodiversity of proteins translated from the limited number of genes in organisms. Inspired by these important biological processes, a strong driving force is to develop efficient peptide and protein bioconjugation methods for mimicking and expanding the scope of the PTMs to create novel bioconjugates as reported in literature reviews [9,10,11,12,13,14,15,16,17,18,19,20].

Peptide and protein modification has been used for various applications such as medical diagnostics (fluorescent labels) [21,22], biopharmaceutical conjugates (PEGylation, lipidation, antibody–drug conjugates, vaccines, and drug delivery) [23,24,25,26,27,28], bioimaging (targeted imaging agents) [29,30], biosensors [31,32], biological tools to study cellular pathways and molecular mechanisms [23,33], biocatalysts [34,35], and materials science [36,37,38,39,40]. However, there are many challenges in peptide and protein modification including chemo- and regioselectivity, functional group compatibility, mild conditions, aqueous media, physiological conditions (pH 6–8, <40 °C, and <0.1% organic solvents), short reaction time, low reagent concentration, nontoxic reagents, high yield, scalability, generally applicable with proteins, and simple purification to retain the integrity of bioactivity and stability of bioconjugates without undesirable protein denaturation and aggregation.

**Figure 1:**
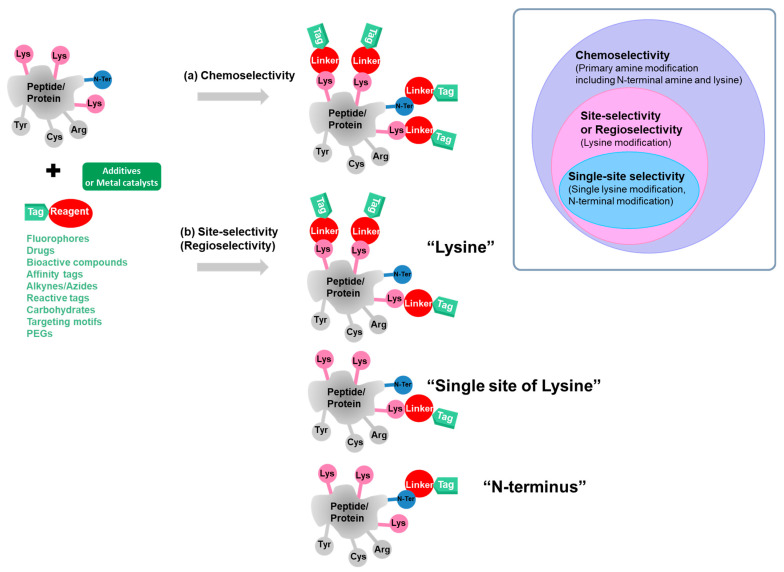
The classification of selectivity of primary amine modification for peptides and proteins presented in this review includes (**a**) chemoselectivity, (**b**) site-selectivity or regioselectivity for lysine, single site of lysine, and N-terminus. Inset: the classification of bioconjugation selectivity.

Given the diversity of amino acids in peptides and proteins, selective modification is of great importance to reduce the possibility of obtaining heterogeneous and undesirable mixtures of bioconjugates. In general, there are two types of selectivity, i.e., chemoselectivity and site-selectivity (or regioselectivity) [19]. Chemoselectivity is used to describe the capability of the bioconjugation reagents to react with one type of amino acid side chain functional group among others (-SH, -NH_2_, and -OH) in the same peptide chain or protein. Site-selectivity (or regioselectivity) is achieved when the bioconjugation reagents selectively react with the same type of functional groups of amino acids existing in different positions (e.g., lysine amino group in different positions, lysine amino group versus N-terminal amino group) (Figure 1). To achieve the selectivity, the chemical reactivity of the functional groups on the bioconjugation reagents and the desirable amino acid residues need to be considered, such as nucleophilicity, hardness/softness, acidity/basicity, and oxidation state. Another important factor influencing the selectivity and is difficult to be predicted is the microenvironment of the proteins resulting from protein folding. Protein folding causes individual interactions in each region of the proteins such as charge–charge interactions, hydrogen bonds, and desolvation leading to different p*K*_a_ among the same type of amino acid residues in different locations [35,41].

Herein, we focus on the endeavors in the modification of primary amines on lysine side chains (ε-amino group) and N-terminus (α-amino group). Lysine is one of the most commonly targeted residues for protein bioconjugation because of its good nucleophilicity and high abundance on proteins and constructed antibodies, and high occurrence on solvent-exposed surfaces of tertiary protein structures. The N-Terminal α-amino group presenting at the N-terminus of proteins is a promising target for the construction of homogeneous bioconjugates. In this review, we outline the content according to the selectivity classified as chemoselectivity and site-selectivity (regioselectivity) (Figure 1). As shown in Figure 1, the bioconjugation reagents (represented by a red circle) attached to the tags (a light green tag) have been used for the bioconjugation to react with the primary amine in peptides or proteins. Some bioconjugation methods require additives or metal catalysts (represented by a dark green rectangle) to promote the conversion. Upon the bioconjugation, the bioconjugates are formed comprising the linker (contributing from the reagents, represented by red color) connected between the tags and primary amine on peptides or proteins. The tags include fluorophores, drugs, bioactive compounds, affinity tags, alkynes/azides, reactive tags, carbohydrates, targeting motifs, and PEGs for further applications. Due to the diversity of proteins, the selection criteria of the bioconjugation reagents are based on their ability to retain or create the desired properties in the resulting bioconjugates. Thus, in this review, we aim to provide useful information for guiding the selection of appropriate bioconjugation methods for different applications.

## 2. Chemoselective Methods for Primary Amine Modification

Primary amines including the ε-amine of lysine and α-amine at the N-terminus appear abundantly on the protein surface, leading to their high accessibility for bioconjugation [42]. The modification of primary amines is one of the earliest and most commonly used methods for bioconjugation [43]. The modification of primary amines is usually achieved by attaching the peptides and proteins with desired functional groups prior to subsequent bioorthogonal reactions (e.g., click chemistry). Here, we classify the modification of primary amines as classical and new methods.

### 2.1. Classical Methods for Primary Amine Modification

**Figure 2:**
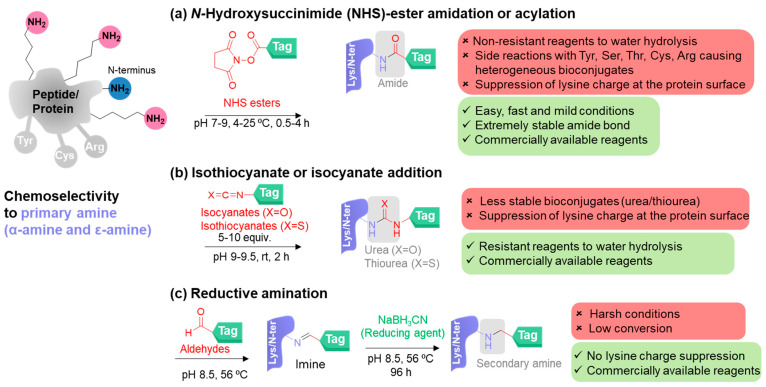
Classical methods for chemoselective modification of primary amines on peptides/proteins. (**a**) *N*-hydroxysuccinimide (NHS)-ester amidation or acylation by using NHS esters, (**b**) isothiocyanate or isocyanate addition by using isocyanates and isothiocyanates, and (**c**) reductive amination by using aldehydes and NaBH_3_CN.

Classical methods for nucleophilic primary amine bioconjugation rely on electrophilic reagents including *N*-hydroxysuccinimide (NHS) esters, iso(thio)cyanates, and reductive amination reactions with aldehydes (Figure 2a–c). For clarity purposes, the representative reaction conditions are mainly used for protein modification. The chemical structures of the reagents are drawn in red. The additives or metal catalysts are demonstrated by green color. The particular linkers in the bioconjugates are assigned in the grey region.

*N*-Hydroxysuccinimide (NHS)-activated esters were first used as the reagents for bioconjugation in 1963 by Callahan et al. (Figure 2a) [44,45]. This conventional reagent is commercially available and easy to be synthesized according to the literature procedures. The reagents are highly reactive to give an amide bond in a few hours under mild reaction conditions (neutral to near-neutral pH, room temperature, and various buffers tolerated). The amide bond formed is exceedingly stable and biocompatible but it suppresses the charge at the protein surface, leading to the alteration of the isoelectric point of the proteins. *N*-Hydroxysuccinimide ester reagents are generated from the carboxylic acid-containing molecules, and the resulting reagents provide lower water solubility than the starting molecules. Using sulfo-NHS, the water solubility of the modified biomolecules can be increased by virtue of the charged sulfonate group. NHS esters are easily hydrolyzed by water, in which the hydrolysis rate depends on the pH and temperature. At pH 7 and 4 °C, hydrolysis of NHS esters possesses a half-life of 4–5 h. At a higher pH, the half-life of the NHS esters is shorter (pH 8, 1 h and pH 8.6, 10 min). Thus, the decomposition of these activated NHS ester reagents by hydrolysis in aqueous solutions is unavoidable. However, the reactivity with other amino acid residues including histidine, serine, threonine, and tyrosine accessible on the protein surface is a major disadvantage of NHS esters, leading to mixtures of bioconjugates modified at different positions.

Isocyanates and isothiocyanates were first introduced as the reagents for primary amine modification in 1937 by Todrick and Walker (Figure 2b) [46]. These reagents have been traditionally utilized to transform primary amines into urea and thiourea derivatives, respectively, by amine addition. Although isocyanates and isothiocyanates are much more tolerant toward hydrolysis, the resulting urea and thiourea are less stable than amide bonds. The well-known reagents of this group include fluorescein isothiocyanate (FITC), which has been used for fluorescent labeling of proteins [47]. Like using NHS esters, this bioconjugation method also alters the charge of the amine side chains of lysine that may change the overall charge of the proteins, resulting in potential alteration of the water solubility and other properties of the modified proteins.

Reductive amination is a two-step transformation (Figure 2c) [48]. An aldehyde and the amino group of a lysine residue condense to give an imine (or a Schiff base) that can be subsequently reduced by sodium cyanoborohydride (NaBH_3_CN), leading to the corresponding secondary amine in an irreversible manner. Exploiting the reductive amination strategy helps to avoid the alteration of the protein charges arising from using NHS esters, isocyanates, and isothiocyanates. However, some drawbacks include harsh reaction conditions, low conversion, disulfide bond reduction of proteins by using NaBH_3_CN, and competing reactions with other aldehyde groups.

### 2.2. New Methods for Primary Amine Modification

**Figure 3:**
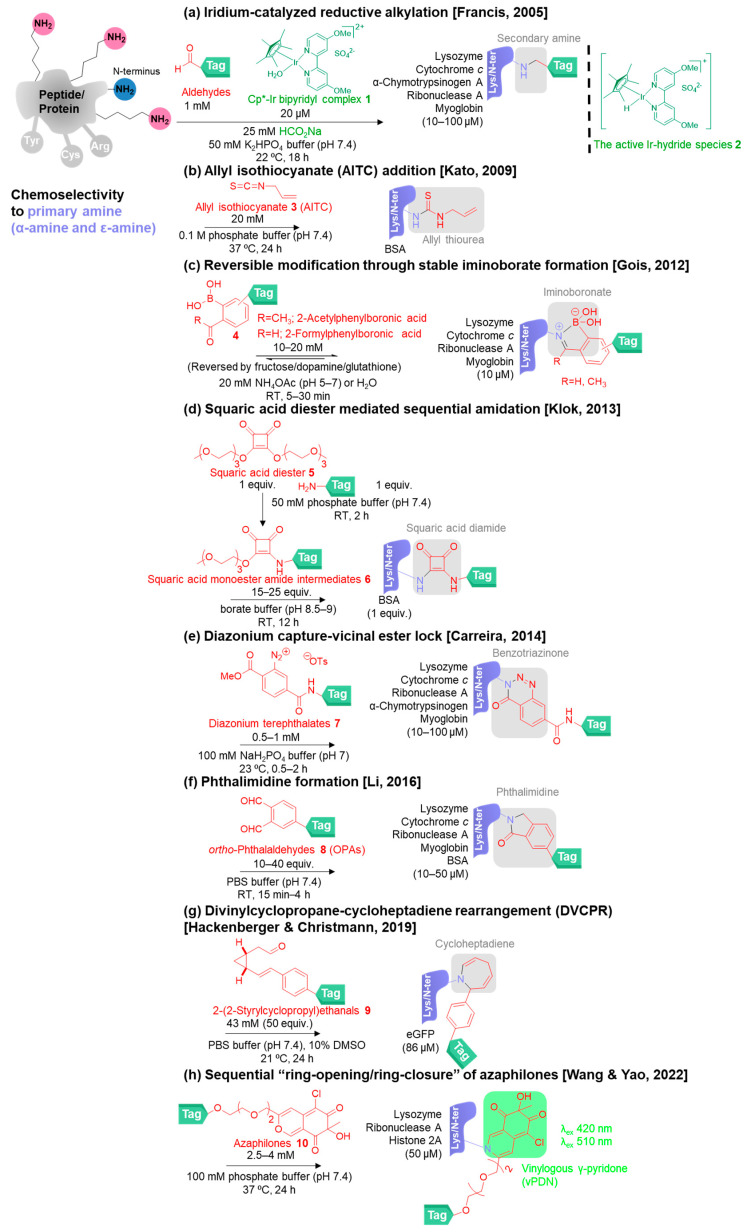
The chemical methods for chemoselective modification of primary amine on peptides/proteins [49,50,51,55,56,57,58,59].

Various chemical approaches have been studied to overcome the limitations of the classical primary amine bioconjugation reactions. To begin with, iridium-catalyzed reductive alkylation of lysine was developed by Francis and co-workers in 2005 (Figure 3a) [49]. The active Ir-hydride species **2** acts as the reducing agent generated in situ by the reaction between sodium formate (HCO_2_Na) and Cp*-Ir bipyridyl complex **1** at physiological pH, showing that this method is milder than the typical reducing agents (NaBH_3_CN). Moreover, it also avoids the use of water-sensitive materials such as NHS esters, allowing the reagents to be more convenient for preparation and storage.

In 2009, Kato and co-workers reported the use of allyl isothiocyanate **3** (AITC; commercially available) for primary amine modification under neutral pH rather than the basic conditions (pH 9) of using isocyanates and isothiocyanates in the classical methods (Figure 3b) [50]. They proposed that the isothiocyanates of AITC reacted rapidly and reversibly with cysteine and subsequently modified lysine and N-terminus to provide the stable allyl thiourea bioconjugates. According to the reaction mechanism, cysteine is required to function as a handle to assist lysine modification in close proximity.

2-Formyl (or acetyl)-phenyl boronic acid (2FPBA or 2APBA; commercially available) reagents **4** were presented by Gois and co-workers in 2012 to label lysine and N-terminus in aqueous media (20 mM NH_4_OAc, pH 7.0) at room temperature providing stable iminoboronates in a short time (5–30 min) (Figure 3c) [51]. The rationale of using boronic acids originated from the reaction between boronic acids and oxygen/nitrogen nucleophiles to form reversible covalent bonds in the boronic acid fabrication of prodrugs, sensors, and hybrid materials [52]. The presence of a formyl or acetyl group at the para position on the 2FPBA or 2APBA reagents allowed the reaction with the primary amine to form imine and induce the formation of the B-N bond on the iminoboranate linkers. This could be the origin of the selective primary amine modification over the hydroxyl group on serine or threonine residues. By using reagents **4**, the reversible modification proceeds based on the formation of a dative B-N linkage which can be converted back to the starting materials via the disruption of the B-N bond by adding fructose, dopamine, or glutathione. The authors mentioned the possibility of using the cleavable linker concept for drug delivery because of the existence of glutathione at the millimolar level in the cell cytoplasm. To apply for in vivo experiments, it is envisaged that the compatibility of the dative B-N linkage with fructose, dopamine, or glutathione in the living systems needs to be considered. Later, Gois and co-workers exploited the use of 2-formyl (or acetyl)-phenyl boronic acids (2FPBA or 2APBA) for further applications involving Gram-positive bacteria labeling and peptide cyclization construction [53,54].

Klok and co-workers introduced squaric acid di(tri(ethylene glycol) monomethylether)ester **5** for primary amine modification via sequential amidation in 2013 (Figure 3d) [55]. First, the intermediate squaric acid monoester amide intermediates **6** were synthesized from the reaction of squaric acid di(tri(ethylene glycol) monomethylether)ester **5** and the tag-linked primary amine in aqueous conditions without adding organic solvent. The intermediate **6** improved the water solubility of the primary amine-linked tag, allowing the use of the organic solvent-free conditions to avoid the denaturation of the proteins of interest in the protein modification step. Then, the proteins were modified by the intermediate **6** to provide the squaric acid diamide bioconjugates.

Another rapid amine bioconjugation (0.5–2 h) was reported by Carreira and co-workers in 2014 [56]. Diazonium terephthalate **7** conjugated to primary amine by a capture-lock approach in which the diazonium captures the amine while the vicinal ester locks it and promotes the intramolecular reaction to give a stable benzotriazinone derivative (Figure 3e).

In 2016, Li and co-workers demonstrated the application of *ortho*-phthalaldehydes **8** (OPAs) for fast and traceless amine bioconjugation in the absence of additives under physiological conditions (Figure 3f) [57]. OPAs are non-hydrolyzable reagents that react with amines rapidly to give phthalimidine bioconjugates.

The use of 2-(2-styrylcyclopropyl)ethanal derivatives **9** as the reagents for targeting amines (including proline) was established by Hackenberger, Christmann, and co-workers (Figure 3g) [58]. The reaction occurred through intramolecular divinylcyclopropane-cycloheptadiene rearrangement (DVCPR) and condensation, leading to the irreversible modification to yield the cycloheptadiene bioconjugates.

Recently, in 2022, azaphilones **10** were exploited as primary-amine-selective bioconjugation reagents by Wang, Yao, and co-workers (Figure 3h) [59]. The reaction between azaphilones and primary amines proceeds through a sequential “ring-opening/ring-closure” mechanism to generate stable and fluorescent vinylogous γ-pyridone (vPDN) moieties with water molecules as the only byproduct.

## 3. Site-Selective Methods for Primary Amine Modification

Site-selective modification is the key to overcome the undesirable formation of heterogeneous bioconjugates and protein mixtures, difficulty in protein purification, and protein functional disruption. Especially from the therapeutic protein point of view, site-selective modification is very important to improve the stability and therapeutic activity by specific amino acid modification or modification of the non-active site amino acids [60,61]. Herein, we divide the site-selectivity or regioselectivity into three types, including site-selective lysine modification, single-site-selective lysine modification, and N-terminal modification [62,63].

### 3.1. Site-Selective Lysine Modification

**Figure 4:**
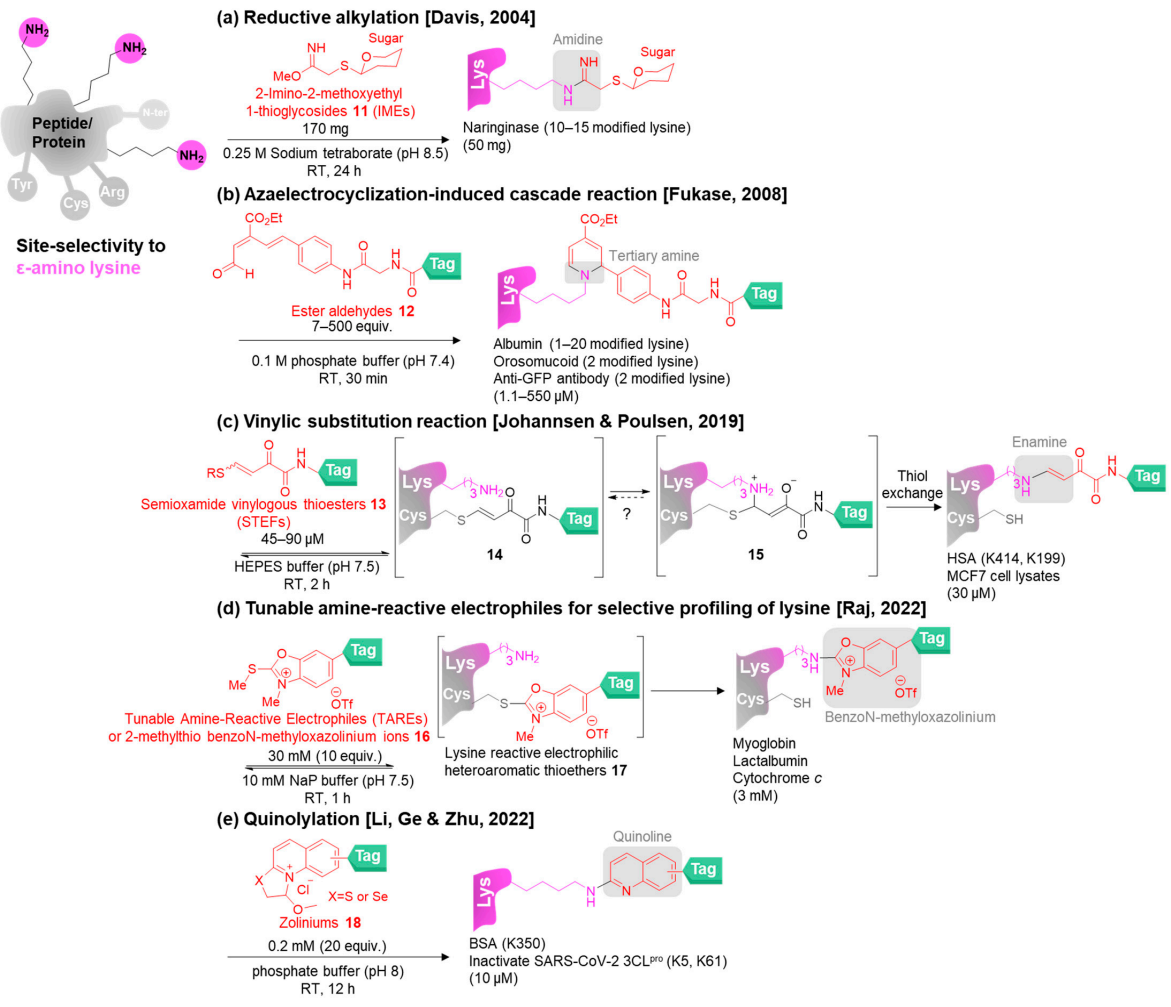
The chemical methods for site-selective modification of lysine on peptides/proteins [69,70,74,75,76].

Lysine is one of the most prevalent amino acids in the human proteome and participates in protein–protein interactions, enzymatic activity, and post-translational modification, suggesting that lysine is a promising target to modulate protein function through modifying lysine residues [64,65]. In particular, lysine modification has tremendous importance in diverse biomedical applications. Given the different p*K*_a_ of primary amines on lysine side chains (N^α^-NH_2_, 10) and N-terminus (N^α^-NH_2_, 8), at near-physiological pH, the α-amino group of N-terminus showed higher nucleophilicity than the lysine side chain due to the protonation of the more basic lysine. As a result, bioconjugation at near-physiological pH is suitable to modify the N-terminal α-amine, while the pH range of 8.5–9.5 provides the optimal pH for lysine modification [11]. Moreover, N-terminal α-amine is generally located on the protein surface and exposed to the solvents, leading to convenient modification [66]. It is also noted that the microenvironment of peptides and proteins is also a major factor to vary the p*K*_a_ of lysine in different positions on tertiary protein structures [67]. Thus, it is a great challenge to label lysine at physiological pH without labeling the N-terminus. We list the lysine-targeting reagents to distinguish the modification of α-amino groups of lysine among other functional groups (α-amino, hydroxyl, thiol), i.e., chemoselective and site-selective modification of lysine (Figure 4).

2-Imino-2-methoxyethyl thioglycosides or IME-thioglycosides were discovered in 1976 as the reagents for immobilizing sugars to proteins by Lee and co-workers [68]. In 2004, Davis and co-workers applied 2-imino-2-methoxyethyl 1-thioglycosides **11** as the lysine-selective reagents to prepare glycoproteins through the formation of an amidine linkage in alkaline buffer (pH 8.5) at room temperature for 24 h (Figure 4a) [69]. The glycoprotein was studied for targeted drug delivery.

Fukase and co-workers developed rapid lysine modification (30 min) through 6π-azaelectrocyclization by using ester aldehydes **12** as the reagents in 2008 (Figure 4b) [70]. They found that the diversity of proteins and reagent concentrations resulted in the different numbers of the labeling molecules on proteins. The large-scale synthesis of the reagents allowed them to launch the lysine labeling kit called “Stella^+^” [71] for molecular imaging [72,73].

In 2019, the acrylate reagents named semioxamide vinylogous thioesters (STEFs) **13** were utilized for rapid lysine bioconjugation (2 h) through a vinylic substitution reaction introduced by Johannsen, Poulsen, and co-workers (Figure 4c) [74]. The authors found that the reagent reacted with the cysteine rapidly in a reversible manner, providing **14,** and then the proximal ε-amino nucleophile attacks the allylic carbon next to the sulfur atom on **14** to provide **15,** leading to the irreversible thiol exchange to obtain the enamine bioconjugate that was assigned as an addition–elimination mechanism. The reagents were not only used for modification of the lysine next to cysteine but also labeled the more reactive lysine residues that were not close to the thiol groups.

The proximity-driven strategy for lysine-selective modification was also established by Raj and co-workers in 2022 (Figure 4d) [75]. The tunable amine-reactive electrophiles (TAREs) or 2-methylthio benzoN-methyloxazolinium ions **16** acted as hydrolysis-resistant and mass-sensitive reagents to provide the stable bioconjugates within 1 h for live cell labeling and proteome profiling. The reagent reacted with the cysteine to generate the lysine reactive electrophilic heteroaromatic thioether **17**. The benzoN-methyloxazolinium linker was obtained after the reaction of lysine and **17**.

Recently, in 2022, Li, Ge, and Zhu and co-workers designed zoliniums **18** possessing good water solubility and low cytotoxicity for quinolylation of lysine residues in pH 8 phosphate buffer (Figure 4e) and showed their applications in peptides, proteins, and living cells [76]. The molecular modeling revealed that the free energy barrier between lysine and the reagent was lower than other amino acids. In addition, the reagents leading to the achievement of chemoselectivity and the site-selectivity were likely to be modified at the hydrophobic or active site.

In general, the preference of lysine modification over N-terminal modification is attributed to the pH control in the experiments taking advantage of the higher p*K*_a_ value of the lysine amino group than the N-terminal amine. When the reactions are conducted at the pH closer to the p*K*_a_ value of the lysine residue, the lysine amino group mainly appears as a free amine available for bioconjugation while the N-terminal amine is protonated.

### 3.2. Single-Site-Selective Lysine Modification

Due to the abundance of lysine on the protein surface, single-site-selective lysine modification is of importance to obtain homogeneous bioconjugates. The homogeneity of the bioconjugates greatly solves the problems of unstable pharmacokinetics and safety. The local microenvironment of proteins is one of the key factors to influence the reactivity of lysine residues. The internal position of lysine in proteins exhibited low p*K*_a_ (~5.3), leading to the unprotonated form at physiological pH and less likely solvent exposure [77]. Thus, the development of efficient reagents for single-site lysine bioconjugation is worthy of pursuit.

**Figure 5:**
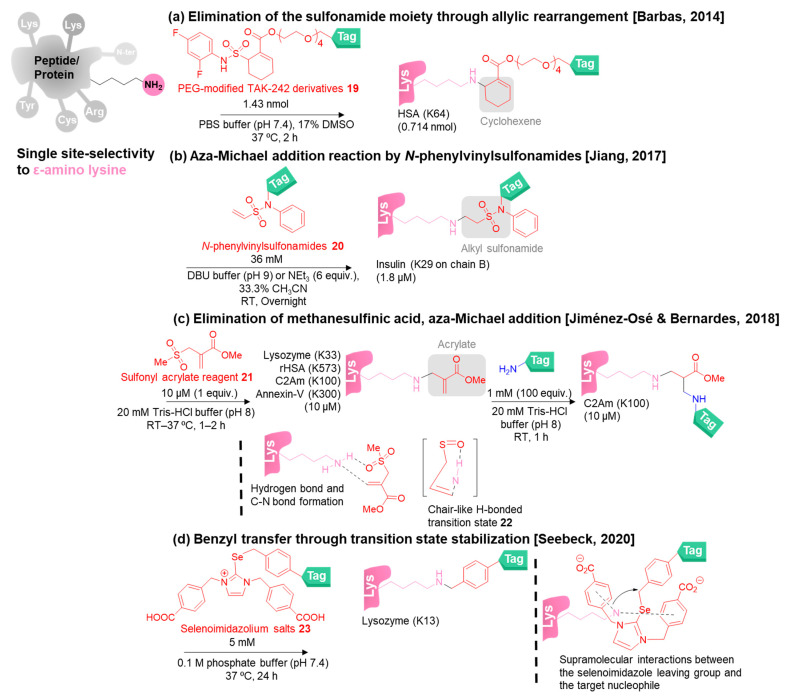

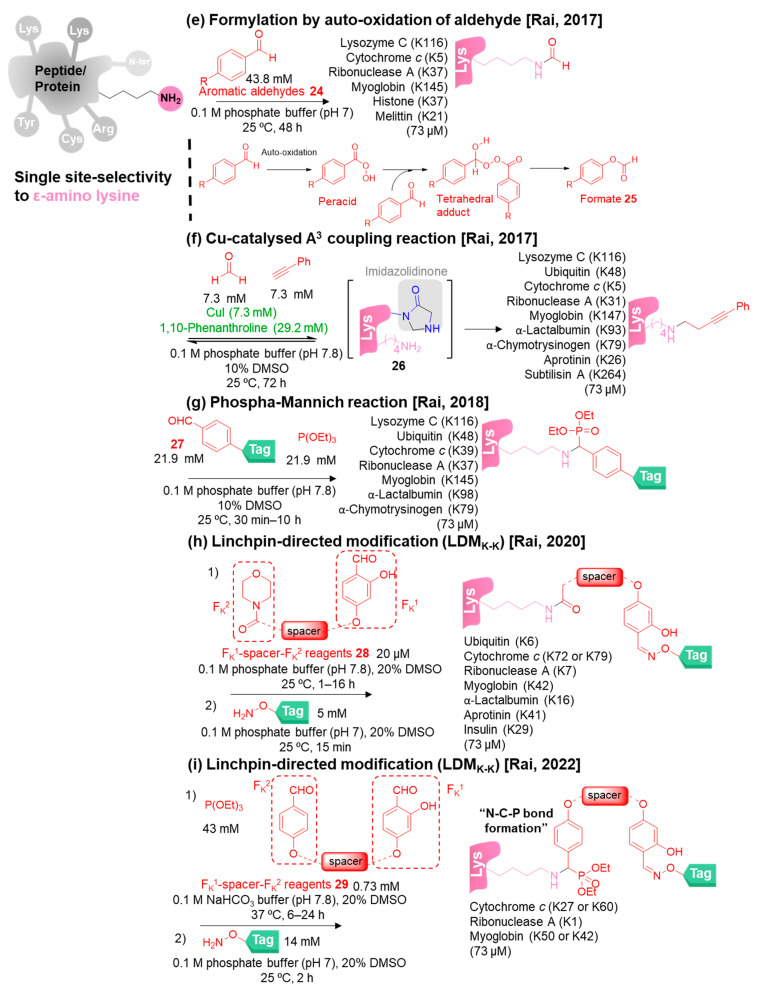
The chemical methods for single-site-selective modification of lysine on peptides/proteins (continue) [78,80,81,82,83,84,85,86,87].

In 2014, Barbas and co-workers devised PEG-modified TAK-242 derivatives **19** as the Michael acceptor reagents to achieve fast single-site lysine modification (2 h) based on addition–elimination of the fluorobenzene sulfonamide moiety through allylic rearrangement to give the cyclohexene linker (Figure 5a) [78]. TAK-242 was known as the Toll-like receptor 4 (TLR4) inhibitor. Using HSA as the model protein, the reagents selectively reacted with the K64 of HSA and generated HSA-cyclohexene conjugates showing significant stability in human plasma and in mice, resulting in improving the therapeutic half-life of the bioconjugates. However, this approach required 17% of DMSO as the co-solvent, which might cause protein denaturation. After that, Barbas and co-workers employed this bioconjugation method for the synthesis of the homogenous antibody conjugate which also used HSA for the bioconjugation [79]. Although this method demonstrated only one model protein as HSA, this method is firstly reported as the regioselective and chemoselective lysine modification.

Next, in 2017, Jiang and co-workers exploited *N*-phenylvinylsulfonamides **20** as the reagents for the aza-Michael addition reaction under basic conditions (DBU buffer at pH 9 or 6 equiv. of triethylamine) in the presence of 33.3% of acetonitrile via an alkyl sulfonamide linkage (Figure 5b) [80]. Using insulin as a protein example, the reagents modified a single site of lysine (K29 on chain B) with excellent specificity.

Similar to reagent **19**, other Michael acceptor reagents based on the addition–elimination reaction mechanism for single lysine modification were subsequently reported by Jiménez-Osé, Bernades, and co-workers in 2018 (Figure 5c) [81]. Sulfonyl acrylate **21** acted as the lysine-selective reagent for the addition–elimination under slightly basic conditions (pH 8) within 2 h to give the acrylate-linked bioconjugates. The computational calculation indicated that the lysine residues with the lowest p*K*_a_ (neutral lysine) could be the most reactive nucleophile to react selectively with the reagent **21**. A chair-like H-bonded transition state **22** was then proposed by the formation of the C-N and hydrogen bonds between the amino group of lysine and the sulfone moiety of reagent **21**, which promotes the aza-Michael addition reaction followed by spontaneous elimination of methanesulfinic acid to provide the acrylate-linked bioconjugates. The acrylate-linked bioconjugates can be applied for further labeling with tag-linked amino compounds through the aza-Michael addition to give the tagged bioconjugates.

In 2020, Seebeck and co-workers reported the use of Se-benzyl selenoimidazolium salts **23** as the new class of electrophilic reagents to proceed with selective *N*-alkylation with the single lysine based on supramolecular interactions between the selenoimidazole leaving group and the target nucleophile (Figure 5d) [82]. The *N*-benzyl side chains of the reagents played important roles in the recognition of the specific lysine via hydrophobic interactions between lysine and the *N*-benzyl side chains, protection of the reagents from hydrolysis, and probably accelerating the benzyl transfer through π interactions. Compared with Michael acceptor reagents, reagents **23** contain the motifs to control the single-site selectivity in which the single-site lysine modification of Michael acceptor reagents could be inherited from the protein local environments.

Rai and co-workers developed various methods for single-site lysine modification involving the formylation by auto-oxidation of aldehyde, Cu-catalysed A^3^ coupling reaction, phospha-Mannich reaction, and linchpin-directed modification (LDMK-K) (Figure 5e–i). All the reagents were designed based on aldehyde reactivity. Various types of proteins were used to show the single lysine labeling.

Firstly, in 2017, they discovered aromatic aldehydes **24** as the reagents for single-site lysine formylation via the auto-oxidation of **24** to form the active formate reagents **25** under physiological conditions for 48 h (Figure 5e) [83]. It took several steps to generate the active formate reagents **25**. Starting from the formation of peracid by auto-oxidation of **24**, it reacted with the resulting peracid to generate a tetrahedral adduct. The rearrangement of the tetrahedral adduct gave the formate **25**, which was further trapped by lysine to yield the formylated bioconjugates. The key to the selectivity between the N-terminal and lysine originated from another task of the aldehyde **24** in transiently blocking the N-terminus. Interestingly, the reagents are able to formylate lysine residue located in the less solvent-accessible position (i.e., α-helix, loop, and β-sheet).

In the same year, they exploited the Cu-catalyzed A^3^ coupling reaction comprising of formaldehyde and phenyl acetylene as the reagents (commercially available), and the Cu–ligand complex (CuI and 1, 10-phenanthroline; commercially available) as a catalyst (Figure 5f) [84]. The transient N-terminal blocking idea was also adopted in this approach, in which formaldehyde reacted reversibly with N-terminal amine and generated the intermediate imidazolidinone **26**, which could be examined by NMR or MALDI-TOF MS. The choice of the ligand (1, 10-phenanthroline) for making the Cu–ligand complex showed an essential role in the selectivity and assisted to inhibit the binding of a Cu catalyst with the protein. Unfortunately, the methods depicted in Figure 5e,f were limited by the variety of the tag on the bioconjugates. Due to the ligand hindering the installation of the chelating functional groups on the reagents, tagging the bioconjugates for other applications was limited. After that, the phospha-Mannich reaction was introduced in 2018 by them to avoid the use of the copper catalyst by addressing the aldehydes **27** and triethylphosphite (P(OEt)_3_) as the reagents (Figure 5g) [85]. In the first step, the aldehydes **27** provided the chemoselectivity to react with lysine to form imine as the latent electrophile. Then, triethylphosphite (P(OEt)_3_) acted as a nucleophile and attacked only one lysine residue with excellent selectivity. This approach allowed further modification to overcome the previous problem of the lack of a convenient site for modification.

The utilization of the aldehyde for lysine masking to promote the single-site selectivity was continued by Rai’s group. In 2020, they devised F_K_^1^-spacer-F_K_^2^ reagents **28** for the linchpin-directed modification (LDM_K-K_) to target the single lysine near another lysine and form the stable amide linker (Figure 5h) [86]. The single-site selectivity can be achieved from the different reactivities of the two electrophilic groups (F_K_^1^ and F_K_^2^) of **28**. Firstly, F_K_^1^ (*o*-hydroxyaldehyde) preferentially reacts with all solvent-exposed lysine residues rapidly and reversibly, providing the linchpin. Secondly, if there is a pair of lysine residues, the nucleophilic lysine close to the linchpin reacts with F_K_^2^ (the activated carbonyl compound by using morpholine as the leaving group) via an intramolecular irreversible reaction in a relatively slow manner and forms the amide bioconjugates. The first step also helps to block the access of the F_K_^2^ to the first lysine. Moreover, the spacer between F_K_^1^ and F_K_^2^ also participated in the site-selectivity. Recently, the second version of F_K_^1^-spacer-F_K_^2^ reagents **29** to form the single lysine modified bioconjugates was reported by the same group (Figure 5i) [87]. Two aldehydes were fabricated as the electrophiles of the reagents. *O*-Hydroxybenzaldehyde served as F_K_^1^, in which it was inactive to P(OEt)_3_, allowing the aldehyde to form the latent imine with all solvent-accessible lysine reversibly and further react with hydroxylamine derivatives for later applications, while the benzaldehyde of F_K_^2^ can react with the P(OEt)_3_ in the presence of the proximal lysine through the phospha-Mannich reaction to achieve the single-site bioconjugates. The different spacers resulted in different modification sites on proteins. Although these methods were highly selective to a single lysine residue, there are some disadvantages, such as no modification site for tagging, long reaction time, catalyst requirement, two-step modification, requirement of a large amount of the reagents, and high content of organic solvents that may require optimization for future applications.

In general, the key to success in the single-site-selective lysine modification could be due to two possible reasons. Firstly, the microenvironment of lysine residues could affect their p*K*_a_ and their solvent accessibility, leading to the reactivity by protonation/deprotonation and accessible modification. Secondly, the fine-tuning of the reagent reactivity as described in Rai’s work promotes the lysine selectivity. The competition between lysine and N-terminal amine selectivity has been commonly controlled by pH adjustment according to their different p*K*_a_ values. At slightly basic pH (around 8), the lysine selectivity will be favored (Figure 5b,c). Although the strategies in Figure 5a,b,d have successfully demonstrated lysine selectivity in proteins, more examples on different proteins would be important to expand the scope of the strategies. Furthermore, computational analysis could provide useful guidance to predict the modification site on the proteins.

### 3.3. Site-Selective N-Terminal Modification

Although various types of chemical probes are able to modify only a single site of lysine among the multiple copies of them, the little alteration of the microenvironment and the chemical structures of the electrophilic probes can dramatically affect the modification sites. Moreover, the difficulties in identifying the precise bioconjugation sites in the high molecular weight proteins could hinder the strategies of single-site lysine modification. The innate single-site modification at the N-terminus helps to overcome these shortcomings from lysine modification. Hence, diverse strategies for chemo- and site-selective modification of the α-amino group have been reported in several reviews [88,89,90,91].

The N-terminal residue in proteins allows single-site modification to prevent the formation of heterogeneous protein mixtures. However, the selectivity between the N-terminal α-amine and abundant lysine ε-amine residues needs to be well-controlled. To facilitate the desirable N-terminal modification, the intrinsic p*K*_a_ differences between the α-amino groups and ε-amino groups at the physiological pH (around 7–7.4) are found to be appropriate for N-terminal modification. Thus, carefully adjusting the pH is a key factor to achieve successful N-terminal modification.

#### 3.3.1. Selective N-Terminal Modification on α-Amino Group

General strategies for targeting the N-terminal α-amino groups of peptides and proteins are elaborated here.

**Figure 6:**
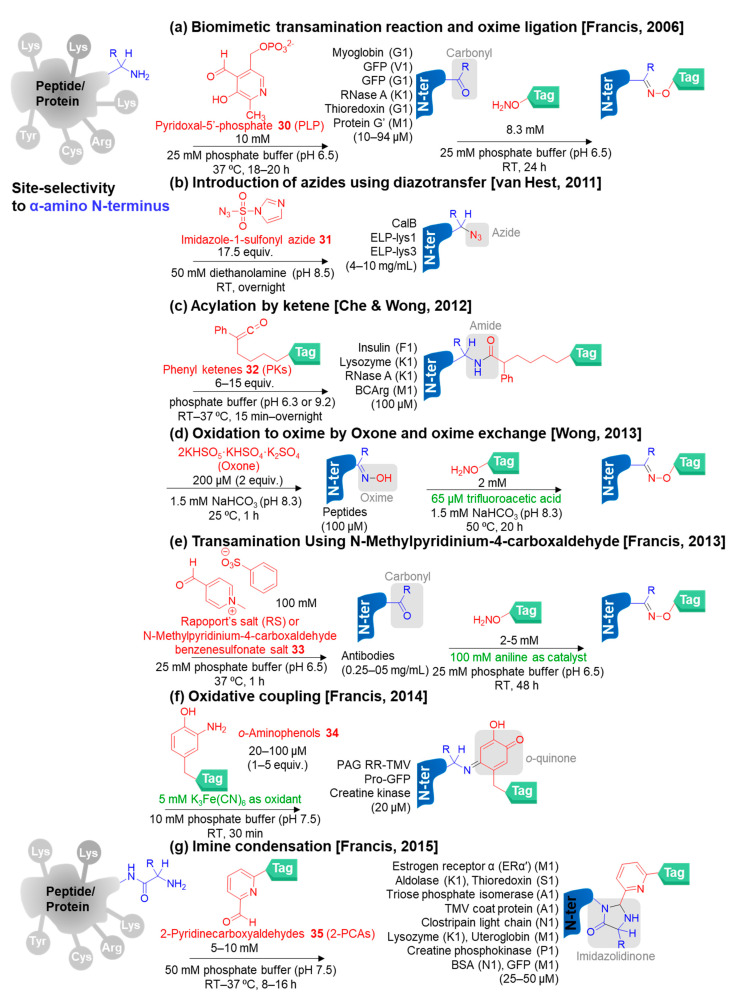

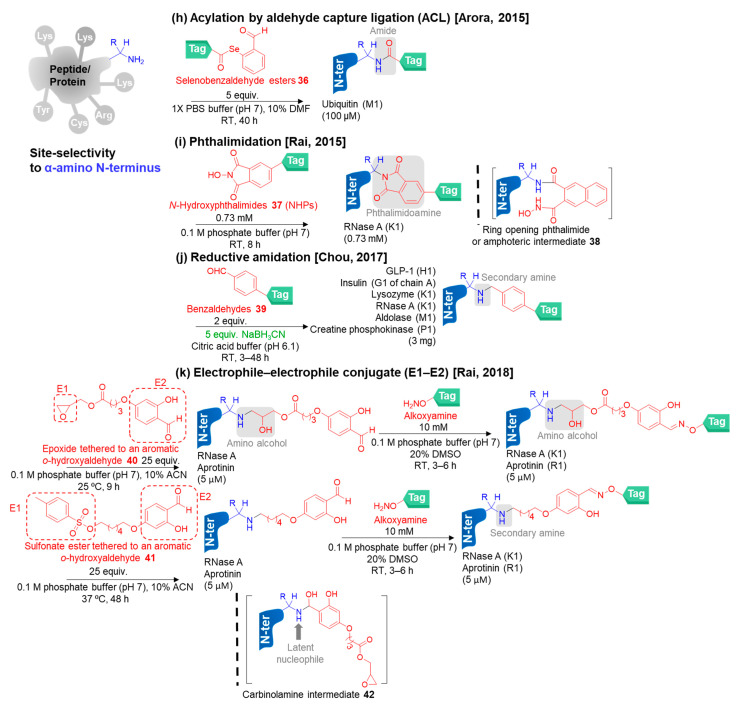

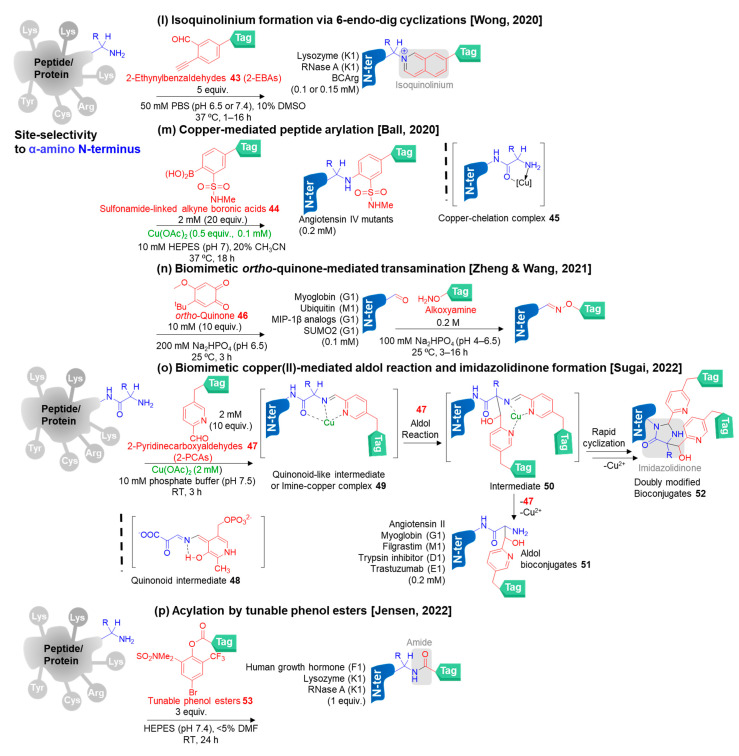
The chemical methods for site-selective modification of N-terminal residue on peptides/proteins [42,92,93,94,95,96,99,100,101,102,103,104,105,106,107,108].

In 2006, Francis and co-workers found that pyridoxal-5′-phosphate **30** (PLP; commercially available) functioned as an efficient reagent for selective oxidation of the protein N-terminus to prepare the carbonyl bioconjugates inspired by a natural process (transamination) (Figure 6a) [92]. PLP is an active form of vitamin B6 acting as the coenzyme that is involved in various enzymatic reactions. Naturally, transamination is catalyzed by PLP-dependent enzymes, in which an aldehyde group on PLP can undergo the condensation reaction with both the α-amino group of N-terminal residue and the ε-amino group of lysine, depending on a particular active site to obtain imine. To achieve the N-terminal selectivity, only imine generated on the N-terminus can be subsequently tautomerized to obtain another form of imine owing to the lower p*K*_a_ of the N-terminal α-amino group (relative to the lysine ε-amino group). The tautomerized imine product allows further hydrolysis to obtain the desired aldehyde or ketone bioconjugates. Afterward, the carbonyl moiety on the resulting bioconjugates provides the valuable synthetic handle to react with alkoxyamine or hydrazide probes through oxime and hydrazone ligation, respectively. The biomimetic transamination reaction by using PLP was successfully modified with the several proteins having N-terminal valine, glycine, lysine, and methionine with moderate to good conversions up to 80%. However, the PLP-mediated transamination reaction was recognized to be incompatible with certain N-terminal amino acids such as serine, threonine or cysteine (because of the possible formation of oxazolidine or thiazolidine ring), tryptophan (because of competing with Pictet–Spengler cyclization), and proline (because of no reactivity).

It was reported in 2011 by van Hest and co-workers that N-terminal α-amine was converted into an azide by using imidazole-1-sulfonyl azide **31** (commercially available) as a diazo transfer reagent under mild reaction conditions at pH 8.5 without Cu(II) catalyst addition (Figure 6b) [93]. The reagent **31** successfully transferred an azide group to the N-terminus of *Candida antarctica* lipase B (CalB) and Elastin-like polypeptide (ELP), but the lysine modification was still observed at a very low level. Importantly, the azide installation is required for click-chemistry reactions to react with an alkyne probe. However, this approach cannot modify the N-terminus of lysozyme due to the poor solvent accessibility of the N-terminus as revealed by its crystal structure.

N-Terminal acylation by phenyl ketene reagents **32** (PKs) was demonstrated by Che, Wong, and co-workers in 2012 (Figure 6c) [94]. Ketenes modified the N-terminus of insulin, lysozyme, RNaseA, and BCArg selectively under conditions of pH 6.3 (room temperature) or 9.2 (37 °C). However, isolated ketenes are difficult to prepare and handle because of their instability.

In 2013, Wong and co-workers reported the use of commercially available oxone (2KHSO_5_·KHSO_4_·K_2_SO_4_) for convenient oxidation of the N-terminal α-amine of peptides to form oxime bioconjugates (Figure 6d) [95]. The resulting oximes showed ability to be further functionalized via transoximation with *O*-substituted hydroxylamines. However, due to the over-oxidation with the thioether group of methionine, thiol group of cysteine, and indole of tryptophan, these drawbacks limited this reagent for N-terminal protein modification.

In the same year, Francis and co-workers discovered *N*-methylpyridinium-4-carboxyaldehyde benzenesulfonate salt **33** (Rapoport’s salt or RS; commercially available) as a new reagent in addition to PLP (Figure 6e) [96]. RS is a readily scale-up and inexpensive bench-top reagent showing higher reactivity than PLP. RS successfully modified the heavy-chain N-terminal glutamates instead of the light-chain N-terminal aspartate in the anti-HER2 Human IgG1 (Herceptin), while PLP modified the light-chain N-terminal aspartate instead of the heavy-chain N-terminal glutamine in the mouse IgGs [97]. Moreover, combinatorial peptide library screening was performed to identify the first three amino acids of the N-terminus to give the highly efficient N-terminal modification. The results suggested that Glu-Glu-Ser (EES) and Ala-Lys-Thr (AKT) in the N-terminal sequences showed highly selective N-terminal modification by using the RS and PLP, respectively [98]. Genetic engineering strategies to introduce the optimal peptide sequence are required to expand the usages of these transamination reagents for N-terminal protein modification.

In 2014, Francis and co-workers developed a direct and fast protein labeling approach through oxidative coupling reaction in the presence of *o*-aminophenols **34** as the reagents and potassium ferricyanide (K_3_Fe(CN)_6_) as the terminal oxidant, while using PLP and RS reagents showed indirect labeling (Figure 6f) [99]. The afforded *ortho*-quinone bioconjugates tolerated the reducing agents, nucleophiles, and acid or basic pH. This approach was more preferable to modify N-terminal prolyl peptides and proteins to obtain high conversion, but cysteine side chains also reacted with *o*-aminophenols. To avoid the introduction of the optimal amino acid sequences to obtain the N-terminal selectivity, Francis and co-workers introduced 2-pyridinecarboxyaldehydes **35** (2-PCAs) reagents in 2015 (Figure 6g) [100]. 2-PCAs showed good conversion for broad N-terminal amino acids in proteins proceeding via imine condensation reaction to form electrophilic imine and subsequent intramolecular nucleophilic addition by the attacking of the adjacent amide nitrogen backbone, leading to the formation of imidazolidinone bioconjugates. This approach required the incorporation of α-amine and the nitrogen of the amide bond to obtain stable imidazolidinones. As a result, the amino group of lysine side chains cannot provide the imidazolidinones, leading to the high selectivity to the α-amine of the N-terminus. 2-PCAs demonstrated high potential to modify the N-terminus of various proteins including estrogen receptor α (ERα′), aldolase, thioredoxin, triose phosphate isomerase, TMV coat protein, clostripain light chain, lysozyme, creatine phosphokinase, BSA, GFP, and uteroglobin. However, N-terminal acylated proteins and proteins containing proline in the second position (e.g., asparaginase) cannot be modified by using 2-PCAs.

Arora and co-workers developed an aldehyde capture ligation (ACL) based on fundamental ligation techniques for N-terminal acetylation in 2015 (Figure 6h) [101]. The use of selenobenzaldehyde ester compounds **36** as the acylating reagents provided the stable amide linker. Firstly, an *o*-aldehyde group on the reagent condensed with an N-terminal α-amine to form these possible intermediates, including hemiaminal, hemiaminal ester, and imine, in a reversible fashion. Secondly, the acyl shifts of the intermediates promoted the formation of N-terminal acylated products through an intramolecular reaction. They proposed that hemiaminal could be the active intermediates. The aldehyde on the reagent is able to react with any amine, leading to the adoption of pH-controlled conditions (pH 7). ACL showed high tolerance to a variety of N-terminal amino acids. Ubiquitin served as a model protein for ACL-mediated N-terminal modification.

*N*-Hydroxyphthalimide reagents **37** for targeting the α-amine of the N-terminus through phthalimidation were exploited by Rai and co-workers in 2015 (Figure 6i) [102]. Although the phthalimidoamine bioconjugates were stable toward hydrolysis, phthalimidoamine was destabilized in the presence of hydrazine. *N*-Hydroxyphthalimide containing two electrophilic sites allowed the one-pot two-stage transformation. The first stage was the rapid and reversible nucleophilic addition between α-amine and *N*-hydroxyphthalimide to generate the ring-opening phthalimide or amphoteric intermediate **38**. The amphoteric intermediate **38** consisted of one latent nucleophile and one electrophile. Then, the intramolecular nucleophilic addition of the latent nucleophile to the electrophilic carbonyl occurred slowly as the rate-determining step in an irreversible manner. The hydroxyl group of the reagent showed a crucial role in the chemoselectivity by controlling the electrophilicity of the carbonyl via regulating n to p* contributions. They also proposed that the kinetic preferences resulting from high solvent accessibility and lower p*K*_a_ of the N-terminal amino acid contributed to the site-selectivity. RNase A bioconjugation was demonstrated using this method.

In 2017, Chou and co-workers revealed the usage of benzaldehydes **39** and sodium cyanoborohydride (NaBH_3_CN) for N-terminal functionalization via the reductive amination to construct the secondary amine bioconjugates (Figure 6j) [42]. This strategy can apply to most of the N-terminal residues except N-terminal cysteines, which resulted in thiazolidine derivatives. The secondary amine linker helped to preserve the N-terminal positive charge, leading to the fivefold bioactivity enhancement of the insulin bioconjugates.

A similar idea of using *N*-hydroxyphthalimide reagents **37** was reported by the same group, in which the first step is fast and reversible like imine formation, while the second step is slow and irreversible. The first step helps to accelerate the second step and achieve site-selectivity. Based on this idea, in 2018, Rai and co-workers designed the E1-E2 reagents **40** and **41** containing two different reactivity electrophiles involving an aromatic *o*-hydroxyaldehyde (E2) and an epoxide or a sulfonate ester (E1) for N-terminal modification of RNase A and aprotinin (Figure 6k) [103]. By using these E1-E2 reagents, *o*-hydroxyaldehyde (E2) initially reacted with the N-terminal α-amine rapidly and reversibly with the kinetic preference to form the carbinolamine intermediate **42** acting as the latent nucleophile for the latter step. They found that the *ortho* hydroxy group of E2 played a crucial role in the stabilization of the intermediate to support the conversion to the final product. Subsequently, the carbinolamine **42** underwent the intramolecular nucleophilic addition with epoxide or sulfonate ester (E1) to obtain amino alcohol or secondary amine bioconjugate products, respectively, with the aldehyde end. Later, the aldehyde in the products was able to react with hydroxylamine reagents through oxime formation for more applications. The authors pointed that *o*-hydroxyaldehyde can change the intrinsic chemoselectivity preference of epoxides or sulfonate esters to favor the N-terminal α-amine.

In 2020, Wong and co-workers developed 2-ethynylbenzaldehyde derivatives **43** (2-EBAs) as the N-terminal modification reagents via isoquinolinium formation (Figure 6l) [104]. The aldehyde group on **43** was responsible for imine formation. Subsequently, the imine attacked the *ortho* alkyne to yield isoquinolinium via 6-*endo*-*dig* cyclization. The N-terminal site-selectivity was achieved by using 2-EBA with electron-donating and weak electron-withdrawing groups under slightly acidic conditions (pH 6.5) with N-terminal bioconjugation of lysozyme, RNAse A, and BCArg mutant as the examples. Interestingly, the N-terminal-modified BCArg mutant exhibited similar enzymatic and cytotoxic activities to the unmodified one.

In the same year, boronic acid reagents **44** bearing an *ortho*-sulfonamide group were introduced as the N-terminal modification reagents by Ball and co-workers (Figure 6m) [105]. The reagents **44** proceeded through a copper-mediated amine arylation by the formation of a copper-chelation complex **45**, which assisted the success of site-selectivity apart from the p*K*_a_ differences. However, 20–30% organic solvents (acetonitrile, tetrafluoroethylene, or DMSO) were required for dissolving the boronic acid reagents.

Apart from exploiting pyridoxal-5-phosphate **30** (PLP) and *N*-methylpyridinium-4-carboxaldehyde benzenesulfonate salt (Rapoport’s salt) **33** as the transamination reagents developed by Francis and co-workers, *ortho*-quinone **46** was reported to mediate transamination by Zheng, Wang, and co-workers in 2021 (Figure 6n) [106]. This N-terminal modification approach was inspired by *ortho*-quinone cofactors of copper amine oxidases (CuAOs). Generally, quinones are involved in a variety of biological processes because of their reduction ability. In this strategy, quinones acted as the oxidants to oxidize the α-amine of protein N-terminus to generate aldehydes or ketones for further oxime formation. Several proteins were studied including ubiquitin, myoglobin, E3 ubiquitin-protein ligase RNF4 (32–133), fragment and small ubiquitin-related modifier 2 (SUMO2), and a library of macrophage inflammatory protein 1β (MIP-1β) analogs. Interestingly, the bioconjugates of MIP-1β led to a 20-fold increase in their anti-HIV-1 activity. Although the high water solubility of PLP and RS salts allow conducting the bioconjugation without organic co-solvents, the difficulty of removing the reagents from the aqueous medium needs to be addressed. Thus, the utilization of neutral *ortho*-quinone could solve this problem. However, the cross-reactivity with thiol groups would require blocking with 2-nitropiperonyl bromide.

The use of pyridinecarboxaldehyde (2-PCA) analogs **47** for N-terminal bioconjugation to generate imidazolidinone bioconjugates was published by Sugai and co-workers in 2022 (Figure 6o) [107]. The advancement from the previous work reported by Francis’s group was the requirement of Cu(OAc)_2_ due to its biomimetic strategy. The present work mimicked the hydrogen bonding of quinonoid intermediate **48** in the biological activity of (PLP)-dependent L-threonine aldolase by using the quinonoid-like intermediate or the imine–copper complex **49** as the key to successful N-terminal modification. Moreover, the high stability of the resulting metal complex also helped to inhibit transamination that can occur via hydrolysis of the intermediate. Thus, this method required copper as the co-reagent to facilitate the double N-terminal modification. Upon the treatment of **47** and Cu(OAc)_2_, the copper-complex intermediate **49** was formed through the condensation of an aldehyde in 2-PCA and N-terminal α-amine (in which an imine was formed) followed by the chelation of copper(II) ions with nitrogen atoms on the imine and the pyridine and an oxygen atom of the neighbor amide bond. The Lewis acidity of the copper ion induced deprotonation of the α-proton, generating a nucleophilic quinonoid-like intermediate to attack another 2-PCA **47** via aldol reaction and form the intermediate **50**. The intermediate **50** can provide two products involving aldol bioconjugates **51** (by the loss of **47** and Cu^2+^) and doubly modified bioconjugates **52**. To produce bioconjugate **52**, a (pyridine-2-yl)methanol moiety at the α-carbon of **50** accelerated the intramolecular cyclization by the attack of the amide nitrogen at the imine carbon via the Thorpe–Ingold effect to form an imidazolidinone ring followed by the removal of Cu^2+^ by EDTA treatment to finally obtain the doubly modified product **52**. Various types of peptides and proteins were modified with 2-PCA in the presence of Cu(OAc)_2_ and the results implied that the pentapeptides (XRVYI) provided doubly modified bioconjugates as the major products, while the longer peptides and proteins mostly afforded the singly modified products via aldol reactions. However, the uncontrolled number of modifications leading to mixtures of bioconjugates was the disadvantage of this strategy.

In addition to phenyl ketenes **32** (PKs) and selenobenzaldehyde esters **36** for selective N-terminal acylation, tunable phenol esters **53** were utilized as the acetylating reagents under pH 7.3 by Jensen and co-workers in 2022 (Figure 6p) [108]. The other substituents on the phenol esters served as a steric shield for hydrolysis by *o*-sulfonic acid or sulfonamide and the reactivity controller by electron-withdrawing groups in the other *ortho* and *para* positions. Interestingly, tuning pH to 11.3 resulted in selective lysine bioconjugation [109].

#### 3.3.2. Selective N-Terminal Modification on Certain Amino Acid Residues

Various amino acid residues at the N-terminus including cysteine, serine, threonine, tryptophan, proline, and glycine have been modified by different chemical reagents. Each N-terminal amino acid carries a distinct functional group for chemical modification, in which these bioconjugations could offer an additional level of selectivity.

- Cysteine

Given the distinctive reactivity of its 1,2-aminothiol group of N-terminal cysteine, the strategies for selective N-terminal cysteine modification have demonstrated great advancement [90]. Herein, we present the major reagents for N-terminal cysteine labeling based on native chemical ligation (NCL) or amide bond formation by using thioesters, thiazoline formation by using 2-cyanobenzothiazole (CBT) and 2-((alkylthio)(aryl)methylene)malononitrile (TAMM), thiazolidine formation by using benzaldehydes, thiazolidino boronate (TzB) formation by using 2-formyl phenylboronic acids (2-FPBA), and 1,4-thiazepa-5-none formation by using monosubstituted cyclopropenones (CPO). Generally, N-terminal cysteine is rarely found in most of the proteins. Thus, modification at the 1,2-aminothiol group needs prior protein engineering to add the cysteine at the N-terminus of the proteins of interest.

The native chemical ligation reaction (NCL) is a chemo- and regioselective reaction to conjugate the N-terminal cysteine with C-terminal peptide thioester under mild, aqueous, and physiological conditions without the use of additives to form a native peptide bond through transthioesterification and S-to-N acyl exchange. Wieland was first to discover the thioester-based chemistry for the synthesis of small Cys-containing peptides in 1953 [110]. Later, Kent and Tam independently introduced this chemistry as NCL for peptide and protein synthesis [111,112]. NCL not only serves as an appealing strategy for large peptide and protein synthesis but also is adapted for N-terminal cysteine modification [113,114,115]. Moreover, implementing NCL techniques in the presence of other free cysteine residues requires more considerations such as choosing suitable linking positions of the proteins of interest, NCL methods, additives, and reaction conditions as the extra cysteine residue might cause protein dimerization and protein folding impairment [116,117,118,119,120,121].

**Figure 7:**
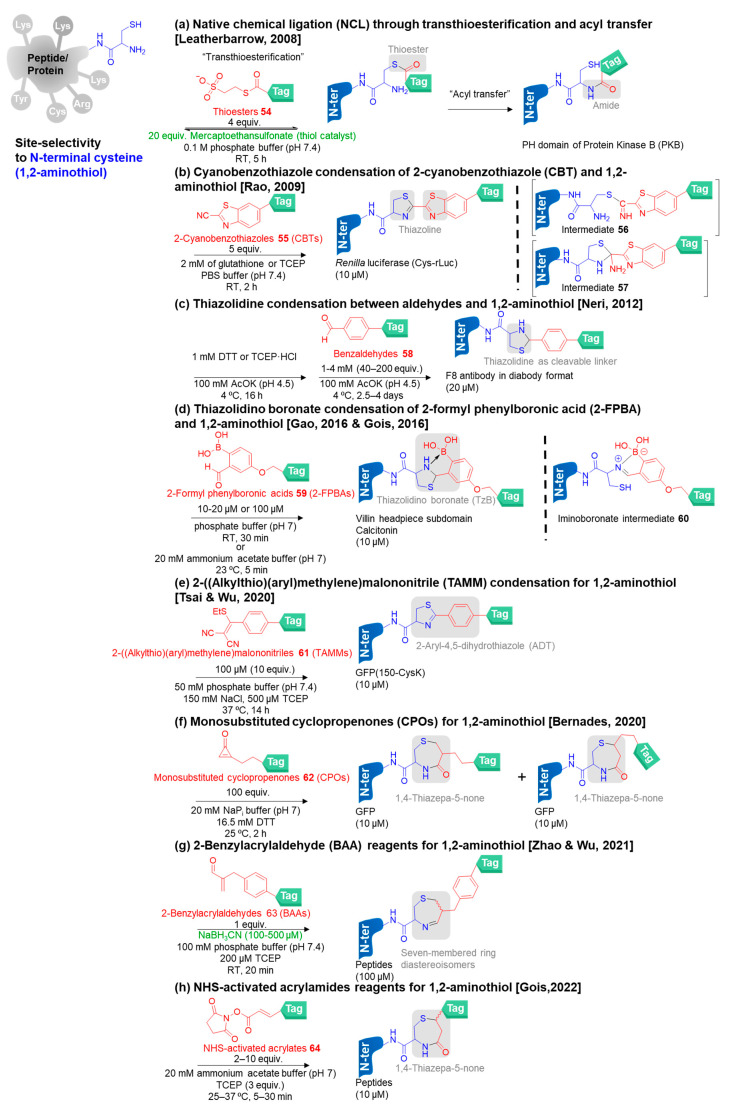
The chemical methods for site-selective modification of N-terminal cysteine on peptides/proteins [122,124,129,133,134,136,137,138,139,140,141].

As native chemical ligation (NCL) requires proteins containing N-terminal cysteine residue, Leatherbarrow and co-workers proposed an enzymatic method to produce N-terminal cysteine protein fragments to react with thioester reagents **54** by using the highly selective cysteine protease named 3C protease (3Cpro) extracted from Foot-and-Mouth Disease Virus (FMDV) (Figure 7a) [122]. Additionally, mercaptoethansulfonate was exploited as the thiol catalyst for the reaction between the N-terminal cysteine and thioester. The detection of membrane-bound phosphatidylinsoitol lipids demonstrated the significance of this protein modification. Although this method is fast and performs at pH 7 at room temperature, the thioester reagents are difficult to prepare and easy to degrade. In 2018, Cole and co-workers adopted the NCL method by using the thioester reagents generated in situ from the reaction between the classical *N*-hydroxysuccinimide (NHS) esters and mercaptoethane sulfonate, namely, MESNA addition [123]. The N-terminal cysteine protein (glutathione S-transferase and uracil DNA glycosylase) containing internal lysine and cysteine showed low levels of off-target labeling (<5% labeling). However, the kinetics are relatively slow (2 to 3 days) as a major drawback.

Rao and co-workers revealed a water-compatible condensation of 2-cyanobenzothiazole derivatives **55** (CBTs) with 1,2- or 1,3-aminothiols for N-terminal cysteine modification in 2009 (Figure 7b) [124]. The reaction resembled the final step of the synthesis of D-luciferin, which is a common substrate for firefly luciferase. The modification proceeded very fast as the second-order rate constant was determined to be 9.19 M^−1^ s^−1^. The application of this work was demonstrated by the labeling of N-terminal cysteine residues on proteins in vitro and on cell surfaces. Later, the mechanistic study of CBT-Cys modification was performed by Liang and co-workers in 2017. The reaction mechanism involved a nucleophilic attack of the thiol group on N-terminal cysteine at the cyanocarbon of the CBTs to form the intermediates **56**. After that, the N atom of 1,2-aminothiol attacked the carbon atom on CBTs to close the ring and form the intermediates **57**. Lastly, the amino group on **57** took a hydrogen atom from the nitrogen on the thiazolidine ring hydrogen to release ammonia gas (NH_3_) and yield the thiazoline bioconjugates [125]. This method has been widely used because of fast modification, good yield, biocompatibility, and stable thiazoline linkage and has been named as a CBT-based click reaction [126]. In 2020, Wood and co-workers used this facile strategy to label antibodies at an N-terminally incorporated cysteine [127]. Later, Gao and co-workers applied CBT-based reagents to target only the α-amine of N-terminal cysteine instead of the 1,2-aminothiol group by adjusting the pH to 8.5 [128]. The free thiol group of the N-terminal cysteine was then modified with a maleimide probe. Hence, this strategy was called “N, S-double labeling”. However, the CBT-based probes have a shortcoming as they react quickly with free thiols in a reversible manner, in which free thiol protection is required.

Formaldehyde has been extensively utilized for discrete residue bioconjugation such as lysine, serine, threonine, and cysteine. In 2012, Neri and co-workers established the use of benzaldehydes **58** for N-terminal cysteine modification to attach the cytotoxic aldehyde drug through thiazolidine formation (Figure 7c) [129]. The reaction mechanism went through an imine formation of the aldehyde and N-terminal α-amine, followed by a nucleophilic addition of the thiol group at the nitrogen atom of imine to obtain the thiazolidine bioconjugates. Importantly, thiazolidines have an advantage as the cleavable linkages release cytotoxic agents in which the stability of the bioconjugates should be suitable for the pharmacokinetics of such an application. However, this method has several drawbacks including a slow reaction rate (2.5–4 days), low pH (pH 4.5), denaturing conditions (1 mM DTT or TCEP·HCl), and an excess amount of the aldehydes (40–200 equiv.) To overcome these drawbacks, the latter N-terminal cysteine modification by using aldehyde reagents to form thiazolidine bioconjugates has been reported by Varghese, Lee, and Li groups [130,131,132].

Gao and Gois groups independently reported another version of aldehyde-based reagents, i.e., 2-formylphenylboronic acids **59** (2-FPBAs) to mediate N-terminal cysteine modification under biocompatible conditions in 2016 (Figure 7d) [133,134]. The modification proceeded rapidly through the iminoboronate intermediate **60** formation to obtain the thiazolidino boronate (TzB) complex bioconjugates with the second-order rate constants of 10^2–^10^3^ M^−1^ s^−1^ at neutral pH. The authors postulated that a boronic acid group at the *ortho* position of benzaldehyde activated the imine to promote thiazolidine formation, leading to the generation of the thiazolidino boronate (TzB) complex. Additionally, an electron-rich boronic acid group could help to accelerate the reaction rate overcoming the slow kinetics resulting from the use of the unsubstituted electron-deficient benzaldehydes used in the previous version. The TzB complex was found to show ready dissociation upon the treatment with benzyl hydroxylamine under mild acidic conditions, providing the possibility of using this method for antibody–drug conjugate construction for drug release in endosomes. However, the thiazolidine in the TzB complex is also dissociated by treatment with free cysteine or hydroxylamine derivatives. To solve the problem of the unstable thiazolidine adducts, in 2020, Gao and co-workers modified the 2-FPBA moiety by introduction of an acetyl ester next to the aldehyde group on the 2-FPBA to produce the acidic stable N-acyl thiazolidines through intramolecular acyl transfer. This pathway could prevent imine reformation and ring-opening, leading to the unstable adducts. The modification of two model proteins, azo-reductase (AzoR) and thioredoxin (Trx) (10 µM), was performed in the presence of the probe (1–5 equiv.) in pH 6 buffer at room temperature, leading to the complete conversions within 2 h. This reagent was also applied to the chemical modification of a phage library [135].

Tsai, Wu, and co-workers reported 2-((alkylthio)(aryl)methylene)malononitriles **61** (TAMMs) as the distinct reagents to target 1,2-aminothiol in N-terminal cysteine residue to form 2-aryl-4,5-dihydrothiazole (ADT) as a stable product through the eccentric mechanism in 2020 (Figure 7e) [136]. Firstly, the thiol group of the 1,2-aminothiol replaced a vinyl sulfide group of TAMM reagents. Secondly, the intramolecular Michael addition of the amino group attacked an allylic carbon to form a thiazolidine. The first two steps were reversible. Finally, the thiazolidine was deprotonated and induced the irreversible elimination of dicyanomethanide to gain thiazoline in the 2-aryl-4,5-dihydrothiazole (ADT). To demonstrate the robustness of this method, TAMM reagents were applied for site-specific labeling of a purified protein in vitro on cell-surface proteins on mammalian cells without disturbing cell viability. Moreover, TAMM derivatives containing a chloroacetyl group were utilized for generating cyclic peptides.

After that, Bernardes and co-workers discovered a selective reaction between biocompatible and stable monosubstituted cyclopropenones **62** (CPOs) and the 1,2-aminothiol of N-terminal cysteine to afford a heterocyclic 1,4-thiazepa-5-none linker under mild conditions in 2020 (Figure 7f) [137,138]. Notably, cyclopropenones showed diverse structural advantages including the aromatic character rendering their stability, ring strain, and high dipole moment, allowing the occurrence of cycloaddition and ring-opening reactions, and α,β-unsaturated ketones acting as electrophiles in 1,2- and 1,4-nucleophilic addition reactions. The reaction mechanism was explored by quantum mechanical (QM) calculations, suggesting that it proceeded through conjugation addition, cyclization, and ring expansion. This strategy provided extremely high selectivity in the presence of internal cysteine residues on the same protein, internal cysteine residues on other proteins, biological thiols, and nucleophilic reagents such as DTT. However, in the addition step of the conjugation, the thiolate nucleophile can react on both sides of the cyclopropenone, leading to two different bioconjugates.

In 2021, Zhao, Wu, and co-workers presented the use of 2-benzylacrylaldehydes **63** (BAAs) in the presence of NaBH_3_CN for fast N-terminal cysteine modification without interference from the internal cysteine and lysine residues (Figure 7g) [139]. The amino group of the 1,2-aminothiol moiety attacked the aldehyde and then the thiol group reacted with alkene rapidly because of the proximity driving force followed by dehydration, providing seven-membered cyclic imine. Upon treatment with NaBH_3_CN, the seven-membered cyclic imine was reduced to afford the stable seven-membered ring diastereoisomeric products.

Then, Gois and co-workers combined the fast kinetic acrylates for cysteine modification and NHS for α-amine modification as the bifunctional NHS-activated acrylamide reagents **64** to label N-terminal cysteine, providing 1,4-thiazepan-5-one linker and staple in-chain lysine and cysteine rapidly (Figure 7h) [140,141]. For the reaction mechanism, the thiol group attacked the Michael acceptor and then the amino group attacked an NHS-activated ester, leading to the cyclization to form 1,4-thiazepan-5-one.

- Other specific N-terminal amino acids and special sequences at the N-terminus

N-Terminal tryptophan, proline, serine, and glycine have been specifically modified for peptide and protein bioconjugation including the special sequences at the N-terminus such as histidine tag-glycine.

**Figure 8:**
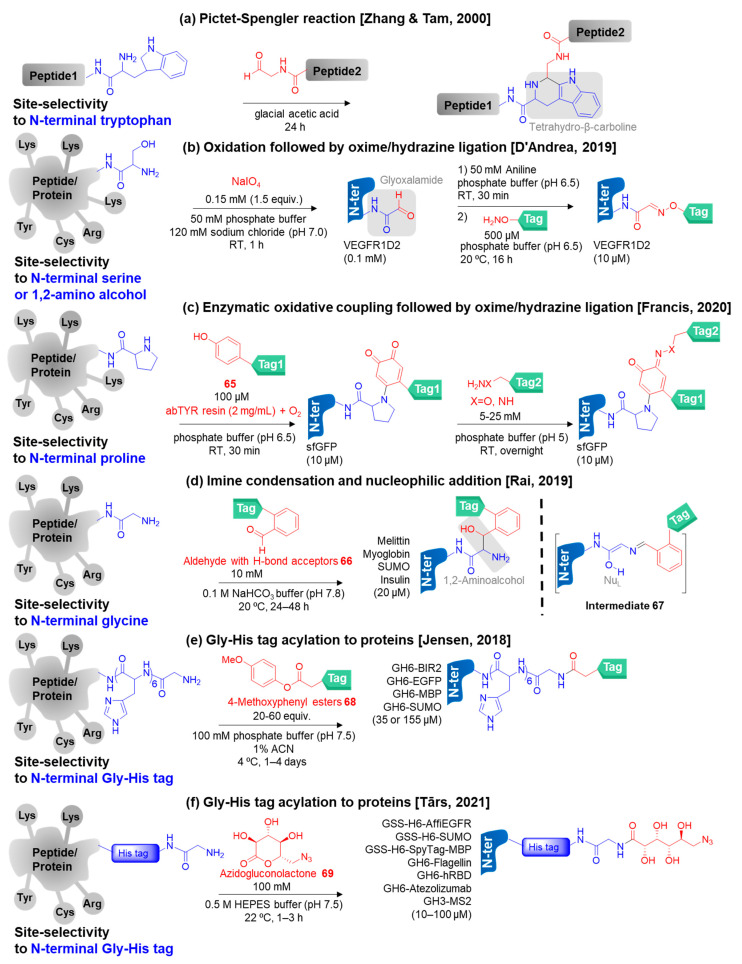
The chemical methods for site-selective modification of other specific N-terminal amino acids (N-terminal serine, proline, and glycine) and special sequences at the N-terminus (N-terminal Gly-His tag) on peptides/proteins [142,144,146,147,149,151].

Zhang, Tam, and co-workers adopted the Pictet–Spengler reaction for a peptide ligation between a peptide containing N-terminal tryptophan and another peptide with a C-terminal aldehyde to provide a distinct tetrahydro-β-carboline linker in 2000 (Figure 8a) [142]. However, this reaction requires acid conditions and is not compatible with native proteins.

N-Terminal serine modification using commercially available NaIO_4_ was pioneered by Geoghegan and co-workers [143]. In 2019, D’Andrea and co-workers utilized sodium periodate (NaIO_4_) to convert the N-terminal serine or 1,2-amino alcohol to aldehyde or glyoxalamide bioconjugates by using the vascular endothelial growth factor receptor 1 (VEGFR1D2) as the model protein (Figure 8b) [144]. The resulting aldehyde group allows the protein labeling through oxime ligation by treatment with hydroxylamine derivatives for further applications. However, the limitation of using NaIO_4_ is the undesirable products coming from oxidation of other amino acids such as Met and Cys upon prolonged reaction time. So, straightaway purification of the glyoxalamide bioconjugates was required [145].

Recently, Francis and co-workers developed a two-step method to modify N-terminal proline in 2020 (Figure 8c) [146]. Firstly, a ketone group was introduced to the N-terminal proline by the oxidative coupling between the N-terminal proline and **65** catalyzed by the enzyme tyrosinase from *Agaricus bisporus* (abTYR) in the presence of oxygen. Secondly, the ketone was employed for linking an additional tag by an alkoxyamine or hydrazine derivatives through oxime and hydrazone ligation.

Switching to N-terminal glycine modification, Rai and co-workers applied *ortho*-substituted benzaldehydes **66** (H-bond acceptors at *ortho*-position) as the reagents to react with N-terminal glycine specifically in 2019 (Figure 8d) [147]. The imine electrophile (E_L_) was formed from the reaction between the aldehyde reagent and α-amine of the N-terminus as usual through imine condensation. Importantly, they found that only E_L_ generated from N-terminal glycine allowed the formation of the key intermediate **67** possessing a latent nucleophilic N-terminal imine (Nu_L_) with the assistance of H-bond acceptors in the imine group. Then, the Nu_L_ on **67** reacted with the reagent **66** again via nucleophilic addition and dehydration, subsequently leading to the formation of an amino alcohol bioconjugate. Although this approach can be performed under physiological conditions, it required an excess amount of the reagent (500 equiv.) and a long reaction time (24–48 h). Recently, in 2022, the same group expanded the scope of using *ortho*-substituted benzaldehyde **66** reagents by further treatment of the resulting amino alcohol bioconjugates with NaIO_4_ to generate glyoxamide bioconjugates [148]. Then, the glyoxamide bioconjugates allowed the late-stage installation of various tags through oxime ligation.

Moreover, in 2018, Jensen and co-workers employed 4-methoxyphenyl esters **68** as water-soluble acylating agents for N-terminal Gly-His_n_-tagged peptide and protein modification in physiological conditions at 4 °C for 1–4 days (Figure 8e) [149]. The high selectivity of the His tag acylation was obtained through specific base catalysis and proton transfer. The authors proposed that a histidine side chain of the His tag took part in deprotonation during the direct acylation reaction between the glycine α-amine and **68** [150]. After that, Brune, Tārs, and co-workers reported a fast site-selective α-amino acylation for N-terminal Gly-His-tagged proteins by using 6-azido-6-deoxy-glucono-1,5-lactone (6AGDL; commercially available) or azidogluconolactone **69** at neutral pH (7.5) in 2021 (Figure 8f) [151]. Importantly, the reagent **69** was used to install an azide group onto the SARS-CoV-2 receptor-binding domain (RBD) protein antigen. Subsequently, the protein bioconjugates were fabricated onto virus-like particles (VLPs) via strain-promoted alkyne-azide cycloaddition (SPAAC), which was used as virus-neutralizing antibodies in mice. However, the mechanistic studies have not been reported in this work.

## 4. Conclusions

Bioconjugation of peptides and proteins has received significant attention because of its important and diverse applications. In this review, different chemical approaches and reagents targeting primary amines for bioconjugation of peptides and proteins have been summarized and listed according to the chemoselectivity and site-selectivity including their advantages and disadvantages, aiming to facilitate the understanding of each strategy, highlight the selection of a suitable approach for applications, and attract interest on the development of novel reagents for bioconjugation. Although significant advancements have been made in the past decades, most of the bioconjugation methods still need further improvement, such as minimizing the synthetic efforts for reagent preparation, multifunctional linkers for bioconjugation, aqueous conditions around physiological pH without increasing temperature, high degree of selectivity, and fast reaction kinetics, to provide high-quality and versatile bioconjugates in a cost- and time-efficient manner. Given the high proportion of primary amine residues on proteins, the research target for their bioconjugation is to expand the lysine and N-terminal bioconjugation toolbox to be applicable to diverse proteins of interest, broaden the opportunities of the optimal bioconjugation selection, and provide novel bioconjugates with desirable functionalities offering tremendous opportunities in biological, biomedical, and biomaterial applications, for example, the attachment of proteins with various functional units including therapeutic drugs for drug delivery in antibody–drug conjugates (ADCs), fluorophores for visualization and diagnostic imaging, and polyethylene glycol (PEG) for improving pharmacokinetics and pharmacodynamics. Importantly, the advancement of bioorthogonal chemistry and efficient bioconjugation reactions would facilitate the development of more sophisticated and multifunctional bioconjugates with a significant impact on academic research and industrial applications in the coming future.

## Data Availability

Not applicable.
